# Design and Development of Dry Powder Cyclodextrin Complexes of Zinc Diethyldithiocarbamate for Pulmonary Drug Delivery

**DOI:** 10.3390/pharmaceutics18080904

**Published:** 2026-07-23

**Authors:** Ayşe Kaya, Basel Arafat, Havovi Chichger, Barbara Pierscionek, Mohammad Najlah

**Affiliations:** 1 Pharmaceutical Research Group, School of Allied Health, Faculty of Health, Medicine and Social Care, Centre for Biomedicine and Health Innovation, Anglia Ruskin University, Bishops Hall Lane, Chelmsford CM1 1SQ, UK; ayse.kaya@aru.ac.uk (A.K.); barbara.pierscionek@aru.ac.uk (B.P.); 2 Jordan Center for Pharmaceutical Science, Amman 11195, Jordan; b.arafat@jcpr-jo.com; 3 Biomedical Research Group, School of Life Sciences, Anglia Ruskin University, Cambridge CB1 1PT, UK; havovi.chichger@aru.ac.uk

**Keywords:** cyclodextrins, inclusion complexes, pulmonary delivery, zinc diethyldithiocarbamate, lung cancer

## Abstract

**Background:** Pulmonary drug delivery represents a promising approach for the potential localised treatment of respiratory of non-small-cell lung cancer (NSCLC). However, the efficient delivery of poorly water-soluble drugs remains challenging due to limited solubility and inadequate aerodynamic performance. This study aimed to develop and characterise inhalable dry powder formulations of zinc diethyldithiocarbamate (Zn(DDC)_2_) complexes with hydroxypropyl-β-cyclodextrin (HP-β-CD) and sulfobutylether-β-cyclodextrin (SBE-β-CD) for potential pulmonary administration. **Methods:** Formulations were prepared by freeze-drying and spray-drying, with leucine incorporated at 0%, 5%, and 10% *w*/*w*. Formulations were prepared via freeze-drying and spray-drying with leucine incorporation (0%, 5% and 10% *w*/*w*) to evaluate their physicochemical properties, flowability and aerodynamic performance. **Results:** Spray-dried formulations exhibited significantly lower densities (as low as 1.03 ± 0.71 g/cm^3^), enhanced flowability, improved aerosolisation and higher fine particle fraction (FPF) values (up to 40.12 ± 0.60%) compared to freeze-dried powders (20.03 ± 2.79%). The incorporation of leucine further reduced powder density down to 0.72 ± 0.34 g/cm^3^ and increased surface corrugation as shown in SEM images, improving aerosolisation performance, with FPF values up to 76.77 ± 1.18%. Next Generation Impactor (NGI) analysis confirmed that leucine-containing formulations exhibited a greater proportion of particles within the respirable aerodynamic diameter range (1–5 μm), suggesting suitability for deep lung deposition. **Conclusions:** These results demonstrate that spray-dried Zn(DDC)_2_–cyclodextrin powders, particularly those modified with 10% leucine, offer excellent potential for pulmonary delivery in NSCLC therapy. Further in vivo studies are warranted to evaluate therapeutic efficacy and safety.

## 1. Introduction

Lung cancer is a highly invasive and prevalent cancer type, accounting for the primary cause of cancer-related deaths globally. It led to 2.5 million new cases and 1.8 million fatalities [1]. Lung cancer is characterised by tumours originating from the respiratory epithelium and can be classified into two major groups based on pathological distinctions: small-cell lung carcinoma (SCLC) and non-small-cell lung carcinoma (NSCLC) [2]. NSCLC accounts for the majority of lung cancer cases, approximately 85%, while SCLC represents only 15% [2,3].

Despite the advancements in cancer screening and therapeutic treatments, the overall 5-year survival rate for NSCLC remains relatively low, at approximately 26% [3]. This low rate is primarily attributed to the delayed diagnosis of the disease at locally advanced or metastatic stages [4]. Despite chemotherapy being the gold standard therapy for lung cancer treatment, the effectiveness of these cytotoxic agents is limited by their low selectivity and the development of multi-drug resistance. More importantly, their use is often accompanied by severe side effects, which further limit their clinical application [5].

Since the success of inhalable insulin, pulmonary drug delivery has attracted research interest due to its potential for treating chronic diseases through inhalation [6]. Pulmonary drug delivery is considered an ideal approach for treating lung diseases such as pulmonary fibrosis, pneumonia, and lung cancer [5]. In contrast to conventional administration routes, this approach facilitates the efficient delivery of drugs directly to the lungs, by passing first-pass metabolism and thereby minimising systemic adverse effects [5,7]. Additionally, pulmonary drug delivery enhances drug absorption due to the lungs’ unique physiological characteristics, including extensive blood supply, large alveolar surface area, thin alveolar epithelium (~0.2 μm), and low enzymatic metabolism [5,6,7,8].

Despite the advancements in pulmonary drug delivery systems in recent years, the delivery of poorly water-soluble drugs to the lungs with high bioavailability remains a substantial challenge. Undissolved drug particles reaching the lungs may be cleared by the mucociliary escalator in the conducting zone or engulfed by alveolar macrophages in the respiratory zone before exerting their therapeutic effects [7]. Furthermore, insoluble particles can cause lung irritation and adverse effects due to their prolonged retention in the lungs [7]. To enhance solubility and dissolution rate, various strategies have been developed, including amorphous solid dispersion, nanocrystals, and micelles [8,9]. Nevertheless, these approaches have certain limitations, including suboptimal aerodynamic performance and the potential for pulmonary toxicity [10].

Aerodynamic performance plays a pivotal role in pulmonary drug delivery, directly influencing drug deposition in the lungs and, consequently, the overall bioavailability of the drug. For effective lung deposition, inhalable particles should have an optimal aerodynamic diameter ranging from 0.5 to 5 μm; otherwise, they may fail to reach the lungs [7,8,10]. Furthermore, the strict regulations on excipients in pulmonary drug formulations, due to potential pulmonary toxicity, limit formulation development options [10]. Consequently, there is an imperative need to devise a novel strategy that optimises the dissolution rates of poorly water-soluble pharmaceuticals, simultaneously ensuring optimal aerodynamic performance and high biocompatibility.

Cyclodextrin-based formulations have demonstrated significant promise in the treatment of lung cancer [10,11]. They reduce the systemic toxicity by releasing the drug within lung tissues [11]. Furthermore, modified cyclodextrins such as hydroxypropyl-β-cyclodextrin and sulfobutylether-β-cyclodextrin can enhance drug permeation across the lung epithelium while maintaining retention for sustained drug release [10,11,12]. Cyclodextrin complexation has been demonstrated to augment drug transport across the intestinal membrane while simultaneously inhibiting the efflux activity of P-glycoprotein (P-gp), thereby enhancing drug bioavailability and promoting drug accumulation within cancer cells [12]. This mechanism is achieved through the modulation of P-gp and other efflux transporters, effectively circumventing multi-drug resistance (MDR) mechanisms in NSCLC [12].

Metal-containing compounds have been utilised as therapeutic and diagnostic instruments in the treatment of a diverse range of diseases [13]. The efficacy of cisplatin (cis-[PtII(NH_3_)_2_Cl_2_]) in cancer therapy prompted research on employing alternative metal complexes as potential anticancer agents [14]. Bis(diethyldithiocarbamate) zinc (Zn(DDC)_2_), a metabolite of disulfiram (DS), an anti-alcoholism drug, exhibits potent anti-cancer activity in vitro [15]. However, its clinical application is limited due to its poor aqueous solubility [15]. Cyclodextrin complexes incorporating Zn(DDC)_2_ was previously formulated to enhance the solubility of the drug for subsequent applications [16]. The resulting complexes exhibited a substantial improvement in drug solubility, accompanied by an increase in in vitro cytotoxic activity when evaluated against human lung adenocarcinoma cell line A549 [16].

Among the diverse inhaled devices employed for pulmonary delivery, dry powder inhalers (DPIs) are gaining prominence due to their user-friendly nature, compact design, and the enhanced chemical stability of active pharmaceutical ingredients (APIs) in powder form in comparison to their liquid counterparts [17]. In this study, cyclodextrin inclusion complexes of Zn(DDC)_2_ were engineered into a dry powder for pulmonary drug delivery via dry powder inhalers (DPIs). The generated powders were characterised and tested for their aerodynamic size diameter using in vitro simulated lung models for potential treatment of NSCLC via inhalation.

## 2. Materials and Methods

### 2.1. Materials

Zn(DDC)_2_ (>99.0% purity) was purchased from Tokyo Chemical Industry (TCI) Co., Ltd., Tokyo, Japan. 2-hydroxypropyl-beta-cyclodextrin of USP grade (HP-β-CD) (molecular weight: 1555 g/mole) and sulfobutyl ether beta-cyclodextrin sodium (SBE-β-CD) (molecular weight: 2242.05 g/mole) were bought from Glentham, Corsham, UK. MD. l-Leucine (≥98%) was purchased from Sigma-Aldrich (Dorset, UK).

### 2.2. Preparation of Cyclodextrin-Based Powders for Pulmonary Delivery

The Zn(DDC)_2_–HP-β-CD and Zn(DDC)_2_–SBE-β-CD complexes used in this study were selected based on previously reported phase-solubility and solid-state characterisation studies [17]. Both systems exhibited Ap-type phase-solubility behaviour, indicating a non-linear increase in Zn(DDC)_2_ solubilisation with increasing cyclodextrin concentration rather than a single fixed stoichiometric ratio. Complex formation was previously supported by DSC, FTIR, and XRD analyses. Zn(DD)_2_ complexes were quantified using previously reported method [16]. To assess matrix interference, blank formulation matrices containing HP-β-CD or SBE-β-CD, with and without leucine at the highest tested formulation concentration, were prepared and analysed under identical extraction and chromatographic conditions. No interfering peaks were observed at the retention time of Zn(DDC)_2_, confirming the specificity of the method in the presence of cyclodextrins and leucine.

The present work therefore focused on converting these previously characterised complexes into inhalable dry powders and evaluating the effect of drying method and leucine incorporation on aerodynamic performance.

Two distinct drying methods were employed: freeze-drying and spray-drying. These methods were investigated to assess their impact on the physical properties of the powders, including particle density, morphology, flowability, and inhalation characteristics.

#### 2.2.1. Freeze-Drying

Inclusion complexes of Zn(DDC)_2_ with SBE-β-CD and HP-β-CD were frozen at −20 °C for two hours, then kept at −80 °C overnight; cyclodextrin was used as a carrier and cryoprotectant. Samples were then freeze-dried using a Lyovapor L-200 freeze dryer (Buchi Labortechnik, Switzerland) at a pressure of 0.10 mbar for 72 h.

#### 2.2.2. Spray-Drying

Spray-drying was conducted using a B-290 mini spray dryer (Buchi Labortechnik, Flawil, Switzerland) in open-loop mode with compressed air as the drying gas. Cyclodextrins alone served as solubility enhancers and drying matrix. Parameters were as follows: inlet temperature of 120 °C, outlet temperature of ~69 °C, spray gas flow rate of 40 mm, aspirator at 95%, pump at 15%, and nozzle diameter of 3 mm.

Following freeze-drying or spray-drying, all powder formulations were stored in a sealed desiccator at ambient temperature until further characterisation and aerosolisation testing to minimise exposure to moisture.

### 2.3. Incorporation of Leucine with Spray-Dried Powder for Inhalation

To enhance the aerosolisation, properties of spray-dried cyclodextrin-based powders, L-leucine was added to the pre-formed aqueous Zn(DDC)_2_–cyclodextrin complex feed solutions at concentrations of 5% and 10% *w*/*w* relative to the total solid content. The feed solutions were stirred for 10 min before spray-drying under the conditions described in Section 2.2.2. Thus, leucine was incorporated prior to spray-drying and was not blended with the final dry powders.

### 2.4. Powder Characterisation

#### 2.4.1. Density Determination

Bulk and tapped densities were determined as the following, a precisely weighed (m) mass of each formulation was loaded into a graduated cylinder, and the corresponding volume was recorded as the bulk volume (V_b_). Subsequently, the cylinder was tapped for one minute, and the final volume after tapping (V_t_) was recorded. Measurements were conducted in triplicates for each formulation.

The tap density (ρ_t_) and bulk density (ρ_b_) were calculated using the equations below:

ρ_b_ = m/V_b_ (1)

ρ_t_ = m/V_t_ (2)

#### 2.4.2. Powder Flowability

Flowability was measured using a ERWEKA Granulate Tester (ERWEKA GmbH, Langen, Germany). The flowability is determined by measuring the flow time of predefined powder weight using a laser beam. Three distinct outlet nozzles, each with diameters of 10 mm, 15 mm, and 25 mm, were used. Measurements were taken in triplicate for different formulations.

#### 2.4.3. Scanning Electron Microscopy Analysis

Approximately 10 mg of each dry powder sample was affixed to carbon adhesive discs on aluminium stubs and coated with gold using a Bio-Rad SC-500 sputter coater (Leica Microsystems, Wetzlar, Germany) to a thickness of approximately 10 nm. SEM images were obtained using a FEI electron microscope at 5.00 kV after achieving vacuum conditions. Images were collected at a magnifications range of 100–500×.

### 2.5. In Vitro Powder Assessment Using Simulated Lung Models

#### 2.5.1. Two-Stage Twin Impinger (TSI)

The aerodynamic characteristics and aerosol performance of the formulations were assessed using a two-stage twin impinger (TSI) instrument (Copley Scientific Ltd., Nottingham, UK). A flow meter model (DFM3) was employed to control the flow rate to 60 ± 5 L/min via a vacuum pump (LCP5, Copley Scientific Ltd., UK). Prior to testing, the upper and lower stages were filled with 7 mL and 30 mL of distilled water, respectively. A single-dose Hanihaler dry powder inhalation device was utilised to evaluate the aerosol performance of the formulations. Each run involved the manual loading of 10 hard gelatine capsules (size 3) containing (10 ± 0.5 mg) of the powder formulations into the device. The device was then actuated and inserted into a mouthpiece adaptor, and each capsule was run for 4 s. Three replicates were conducted for each formulation. Samples were subsequently collected from the upper and lower compartments into volumetric flasks, and the volumes were adjusted to 25 mL and 50 mL, respectively. Drug content was subsequently analysed using high-performance liquid chromatography (HPLC) [16].

#### 2.5.2. Next Generation Impactor (NGI)

The aerosol properties of the spray-dried and freeze-dried formulations were assessed using the Next Generation Impactor (NGI, Apparatus E) (Copley Scientific Ltd., UK). The air flow rate was calibrated to 60 ± 5 L/min using a vacuum pump. Prior to testing, the flow rate was validated using a flow meter (DFM3, Copley Scientific Ltd., UK). A pre-separator was employed to collect any powder boluses and large non-respirable particles. The central cup of the pre-separator was filled with 15 mL of deionised water. The micro-orifice collector (MOC) stage was replaced with a filter for better collection efficiency of 0.3 μm particles. Powder samples of 10 ± 0.5 mg were precisely weighed and manually loaded into size 3 hard gelatine capsules. Each capsule was individually loaded into the Handihaler dosage chamber. The device was activated and inserted into a mouthpiece adaptor. For each sample, 10 capsules were utilised, drawing the powders into the NGI for 4 s at a rate of 60 L/min. Subsequently, samples were collected from the mouthpiece, induction port, pre-separator, and stages 1 to 8 of the NGI by rinsing with deionised water. The collected samples were then transferred into volumetric flasks for analysis. Drug quantities were determined using the HPLC method [16]. The following parameters were employed for the analysis of NGI results:

Fine particle fraction (FPF): the mass percentage of drug particles with an aerodynamic diameter less than 5 μm.

Respirable fraction (RF): calculated using Equation (3).

Respirable Fraction (%) = (mass of particles deposited in stages 4–7/total amount nebulised) × 100(3)

Emitted Dose (ED): Calculated as the amount of drug recovered from all NGI stages except device and capsule(4)

Fine Particle Dose (FPD): drug recovered from stage 3 to a micro-orifice collector (MOC)(5)

Mass Median Aerodynamic Diameter (MMAD): the diameter at which 50% of aerosols’ masses are greater than or equal to that diameter and 50% are less than or equal to that diameter. It is calculated from the log probability axes of cumulative fraction against the effective cut-off diameter.

Geometric Standard Deviation (GSD): calculated from the same log plot of MMAD using the following equation:

GSD = (d84/d16)^1/2^ (6)

where d84 and d16 define the diameters of which 84% and 16% of aerosol mass are included.

### 2.6. Statistical Analysis

SPSS software (v29.0.1) was used to statistically analysed all parameters using a student *t*-test for independent two groups or one-way analysis of variance (ANOVA), followed by post hoc test where needed, with *p* values scores of * <0.05, ** <0.01, and *** <0.001.

## 3. Results and Discussion

### 3.1. Powder Characterisation

#### 3.1.1. Tapping and Bulk Density

Powder density is a fundamental parameter for assessing the inhalation properties of the formulation, as it influences several critical properties that affect drug delivery efficiency and the performance of the inhalation device. A key requirement for an optimal dry powder inhaler (DPI) formulation is adequate powder flowability, which is essential for consistent drug release from the device, reliable dosing, effective dispersion, capsule filling, and deagglomeration [17]. Powder density plays a crucial role in determining flowability. Lower-density powders generally exhibit improved flow characteristics, facilitating enhanced deposition in the lower respiratory tract [17].

Table 1 presents the bulk and tapped densities of freeze-dried, spray-dried, and leucine-containing powders. The results indicate that spray-dried powders exhibited significantly lower densities compared to freeze-dried formulations. Furthermore, the incorporation of leucine into spray-dried powders led to a further reduction in powder density, with an increase in leucine concentration from 5% to 10% resulting in a more pronounced and significant decrease in density for cyclodextrin-based formulations. Previous studies have shown that the addition of leucine reduces powder density regardless of its concentration [18]. Particularly, dry powders with a bulk density below 0.4 g/cm^3^ are considered optimal for generating aerosols capable of deep lung deposition [19].

#### 3.1.2. Powder Flowability

Optimal flowability is a critical parameter in the development of dry powder inhaler (DPI) formulations, as it directly influences the performance of the inhalation device, including drug dose actuation and the aerosolisation of powders into respirable particles upon inhalation [20]. The flowability of both freeze-dried and spray-dried formulations was assessed using a laser beam method.

As presented in Table 2, the results indicate that freeze-dried powders successfully passed through a 25 mm nozzle; however, no powder was able to pass through the smaller nozzles of 15 mm and 10 mm. In contrast, spray-dried powders exhibited superior flowability, passing through all three nozzles of the granulate tester (25 mm, 15 mm, and 10 mm). Furthermore, spray-dried powders containing 5% and 10% leucine demonstrated significantly improved flowability compared to leucine-free spray-dried powders. The freeze-dried powders were unable to pass through the smaller nozzle apertures, which may be attributed to their porous, low-density, and agglomerated structure. Such a morphology can increase interparticle cohesion and friction, limiting powder movement through narrow apertures. In comparison, spray-dried powders appeared more discrete, while leucine-containing powders exhibited corrugated surfaces that may reduce particle contact area and cohesion. Therefore, the observed differences in nozzle passage are likely associated with differences in particle morphology and packing behaviour. These findings suggest that Zn(DDC)_2_-CD powders incorporating leucine possess enhanced inhalation potential and improved aerosolisation properties relative to their leucine-free formulations. This improvement correlates with the observed reduction in powder density. The inverse relationship between density and flowability is well-documented, as lower-density particles typically experience reduced interparticle cohesion, enhancing flow and dispersion [21].

#### 3.1.3. Scanning Electron Microscopy Analysis

The morphology and distribution of particle shape are critical determinants of drug deposition within various regions of the respiratory tract following inhalation. The aerosolisation performance is influenced not only by particle shape but also by the inhalation device employed [19,22]. Interparticle interactions are influenced by van der Waals forces, which are dependent on factors such as surface, morphology, particle size, shape, and hygroscopicity [19]. Particles with morphologies that minimise contact area and reduce van der Waals forces exhibit a diminished tendency to aggregate, thereby enhancing their dispersion in aerosolised form [19]. The SEM analysis was qualitative and was used to support the interpretation of particle morphology, while aerodynamic particle-size behaviour was assessed quantitatively using NGI.

**Figure 1:**
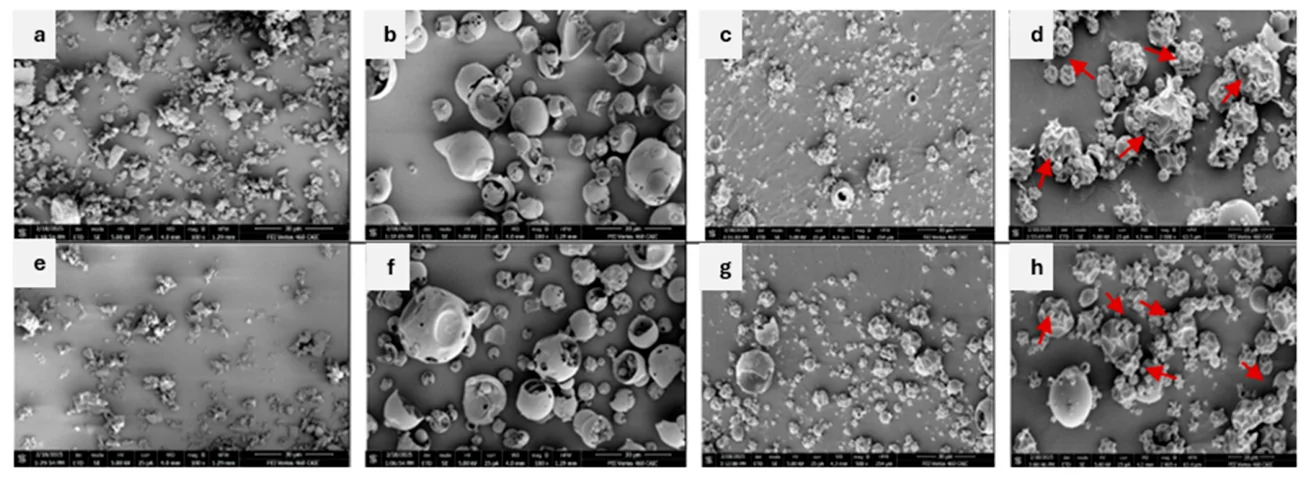
SEM captures of (**a**) SBE-Zn(DDC)_2_ freeze-dried powder; (**b**) SBE-Zn(DDC)_2_ spray-dried powder; (**c**) 5% Leu SBE-SD; (**d**) 10% Leu SBE-SD; (**e**) HP-Zn(DDC)_2_ freeze-dried powder; (**f**) HP-Zn(DDC)_2_ spray-dried powder; (**g**) 5% Leu HP-SD; and (**h**) 10% Leu HP-SD. Red arrows in panels (**d**,**h**) indicate the leucine interlocking effect.

The freeze-dried powder formulations of HP-Zn(DDC)_2_ and SBE-Zn(DDC)_2_ exhibited irregular, elongated morphologies with breadcrumb-like structures, as illustrated in Figure 1a,e. Conversely, spray-dried powder formulations demonstrated spherical, more uniform morphologies with smooth surfaces, as observed in Figure 1b,f. These results align with previous studies, which have shown that freeze-drying results in heterogeneous particle morphologies, whereas spray-drying consistently produces spherical particles [23,24]. Spray-dried powders are generally more suitable for pulmonary delivery than freeze-dried formulations, as elongated particles tend to exhibit stronger interparticle attractive forces, which can negatively impact their aerodynamic performance [24].

Surface roughness is another critical property influencing the behaviour of inhalable powders, as it affects particle packing within powder agglomerates and modulates contact interactions between particles or between particles and the inhaler device [24]. The surface morphology of spray-dried powders was significantly altered upon the incorporation of leucine. The initially smooth, spherical morphology of spray-dried particles transitioned to a wrinkled surface upon leucine addition (Figure 1c,g). This increase in surface roughness is attributed to the formation of a leucine shell around the particles. Moreover, a concentration-dependent effect was observed, where increasing leucine content from 5% to 10% *w*/*w* further enhanced surface roughness. These findings are consistent with previous studies, which reported that leucine incorporation into spray-dried powders resulted in increased surface corrugation [24,25,26].

SEM images revealed the presence of small particles embedded within the grooves of larger particles, as indicated by the red arrows in Figure 1d,h. This interlocking effect was more pronounced at higher leucine concentrations of 10%. It is noteworthy that surface corrugation can have dual effects on powder dispersibility: it may enhance dispersibility by reducing contact points between particles or, conversely, hinder dispersibility due to increased interlocking and entrapment phenomena [26]. Similar observations were reported for disodium cromoglycate, where spray-drying in the presence of leucine resulted in hollow particles encapsulating smaller particles, thereby improving aerosolisation properties. Additionally, leucine incorporation in dry powder inhaler (DPI) formulations for biomacromolecules resulted in particles with wrinkled surfaces and embedded smaller particles, which enhanced their aerosolisation characteristics [26,27]. In this study, the interlocking appeared to aid dispersion, as supported by improved performance in NGI and TSI analyses.

#### 3.1.4. Two-Stage Twin Impinger (TSI)

**Figure 2:**
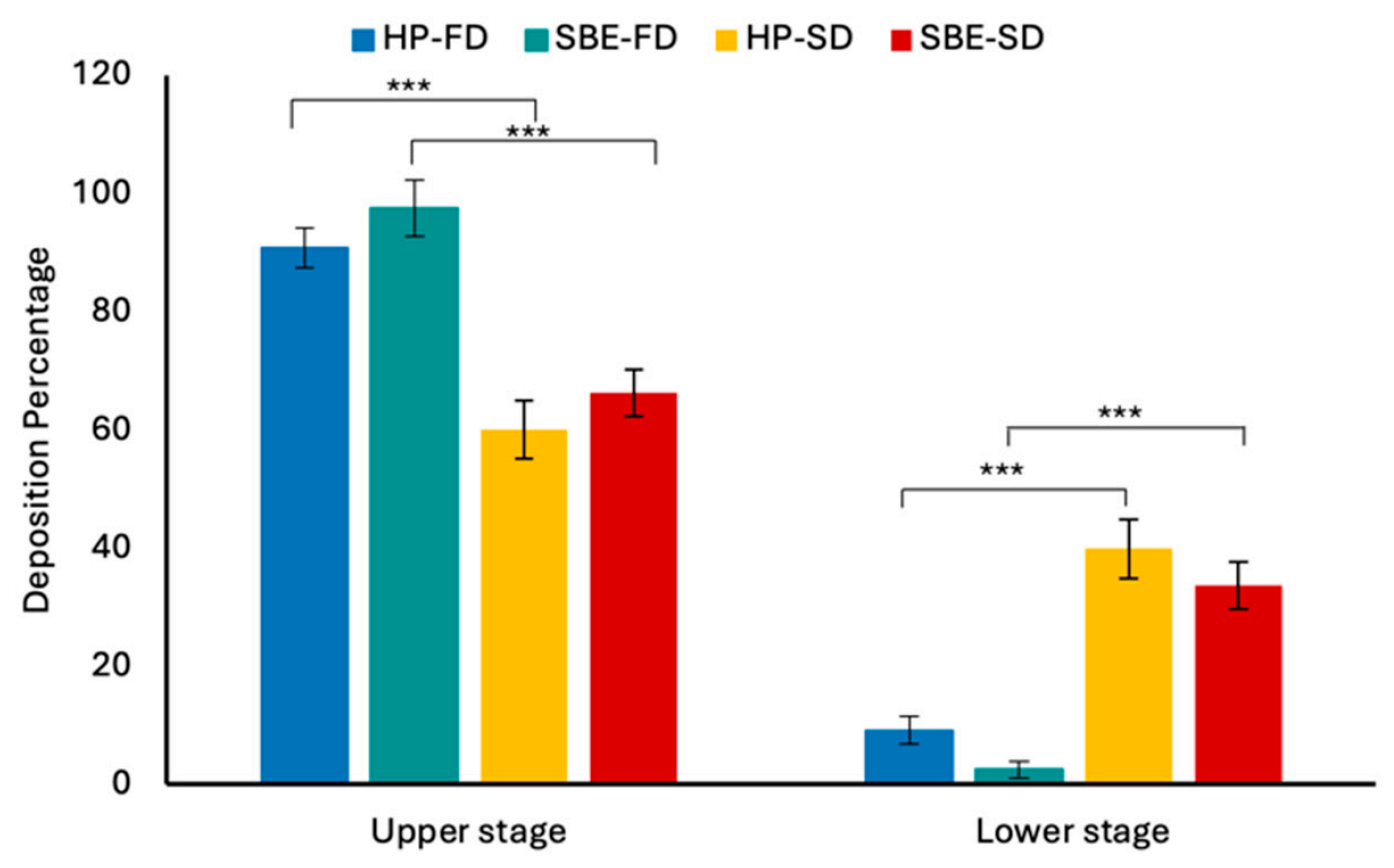
Deposition percentages of HP-FD, SBE-FD, HP-SD, and SBE-SD powder formulations across TSI stages at 60 L/min (mean ± SD, n = 3). *** *p* < 0.001.

The in vitro aerosolisation efficiency of freeze-dried and spray-dried formulations of CD-Zn(DDC)_2_ was evaluated using the two-stage twin impinger (British Pharmacopoeia, Apparatus A, London, UK). As shown in Figure 2, freeze-dried powders primarily deposited in the upper compartment, with 90.7 ± 1.3% and 97.5 ± 0.98% of HP-FD and SBE-FD powders, respectively. However, only 9.20 ± 1.3% and 2.49 ± 0.98% of powders deposited in the lower compartment, which represents respirable fractions.

In contrast, the spray-dried formulations exhibited a statistically significant enhancement in powder deposition within the lower compartment of the TSI, with 39.91 ± 4.9% of HP-SD and 33.69 ± 3.94% of SBE-SD powders accumulating in the respirable stage. This finding suggests that spray-drying yielded powders with a reduced aerodynamic diameter relative to freeze-dried powders, enhancing their potential for pulmonary deposition.

**Figure 3:**
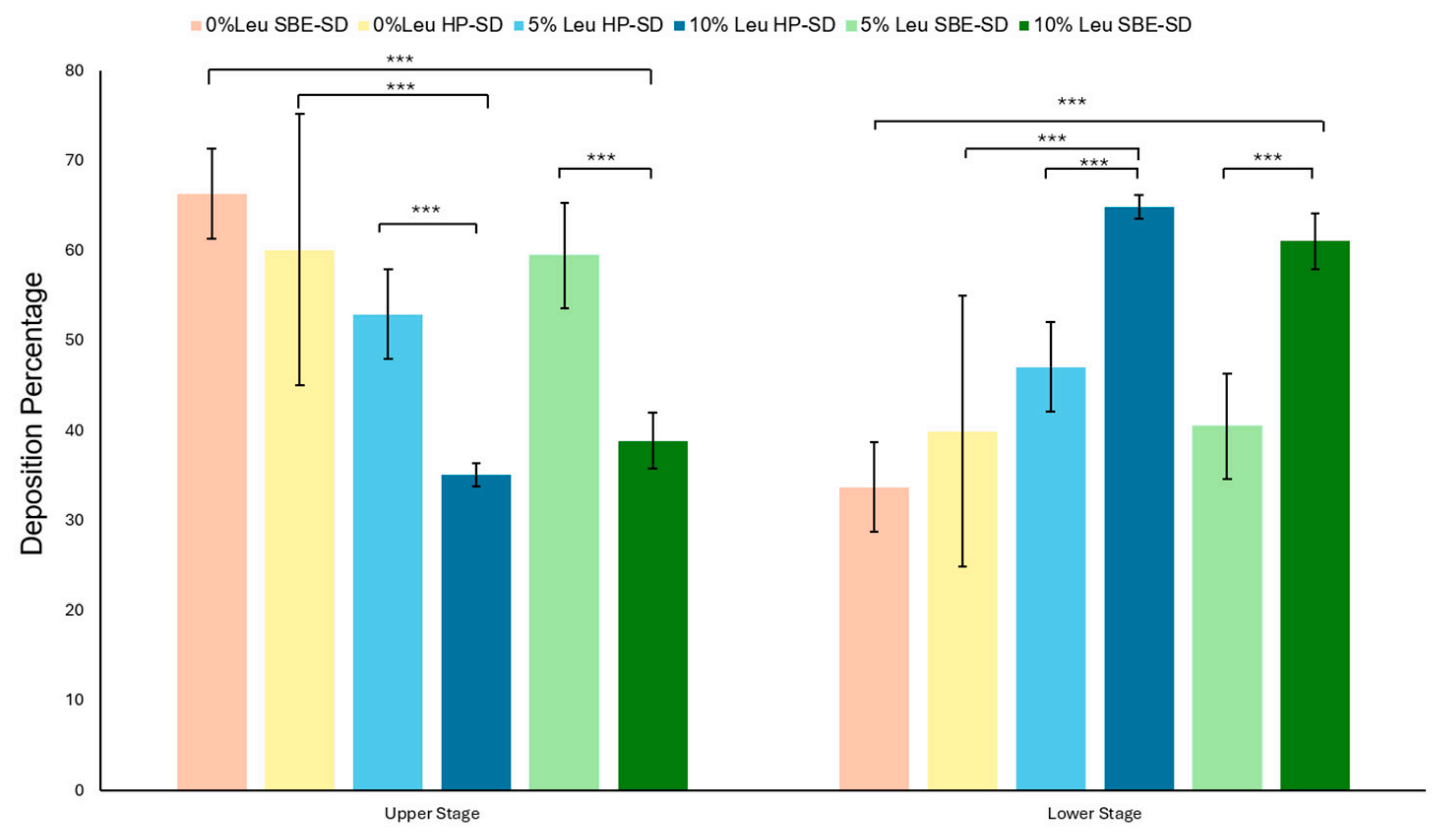
Deposition percentages of leucine-free 5% Leu HP-SD, 10% Leu HP-SD, 5% Leu SBE-SD, and 10% Leu SBE-SD formulations across TSI stages at 60 L/min (mean ± SD, n = 3). *** *p* < 0.001.

Figure 3 illustrates the deposition patterns of spray-dried powders incorporating 0%, 5%, and 10% leucine across the TSI stages. The inclusion of leucine in spray-dried formulations significantly improved deposition in the lower stage of the TSI when compared to leucine-free powders. Notably, an increase in leucine concentration from 5% to 10% further facilitated deposition within the lower compartment, with a significant improvement from 47.06 ± 2.44% to 64.89 ± 1.26% for HP formulations and from 40.50 ± 5.86% to 61.11 ± 3.10% for SBE-CD formulations. This enhancement may be attributed to the physicochemical properties of leucine, forming hollow and wrinkled particles with reduced bulk density, thereby improving aerosolisation efficiency.

#### 3.1.5. Next Generation Impactor (NGI)

**Figure 4:**
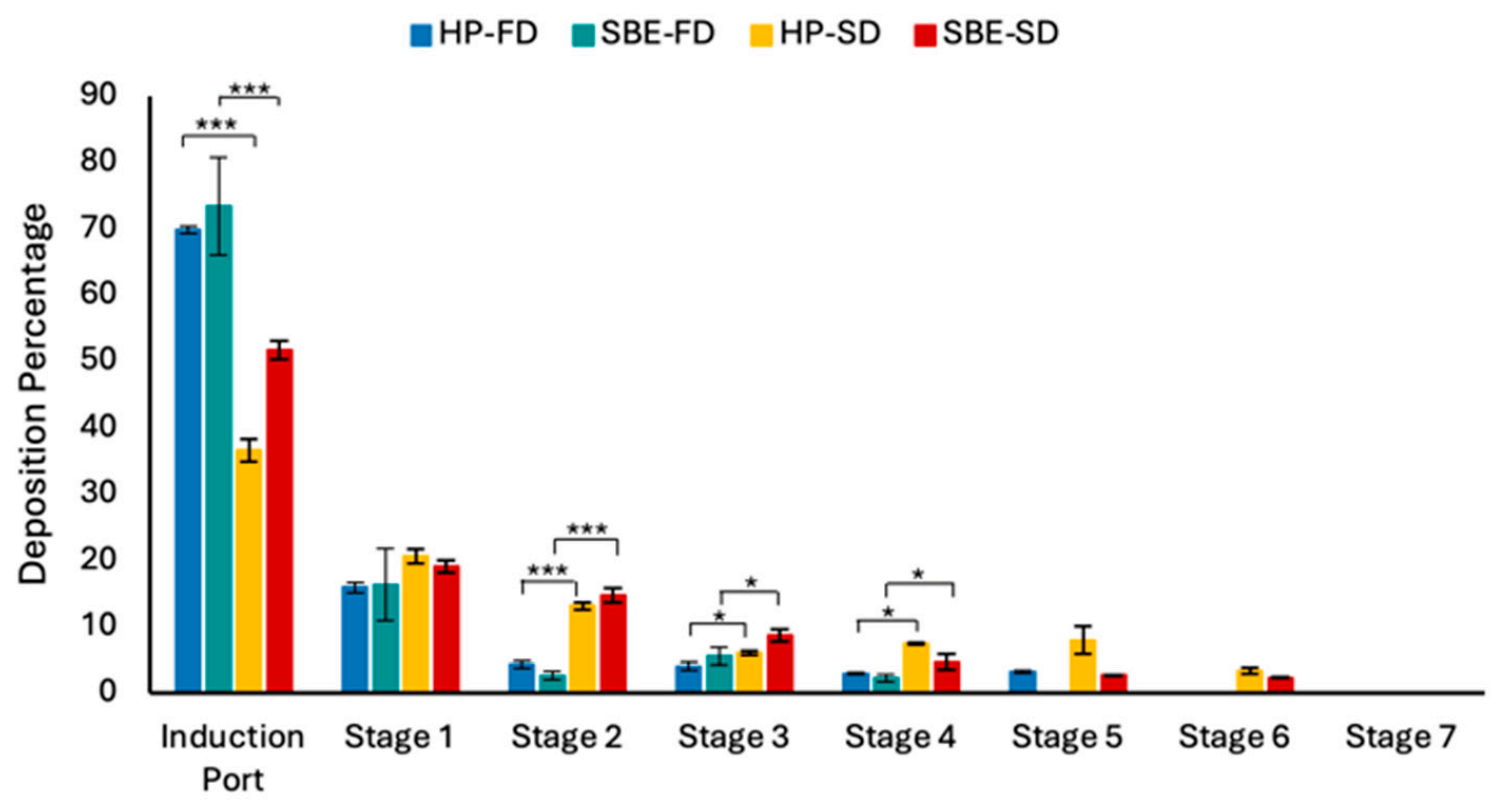
Deposition percentages of freeze-dried and spray-dried powders across NGI stages at 60 L/min (mean ± SD, n = 3). * *p* < 0.05; *** *p* < 0.001.

The in vitro deposition profile of the powder formulations was further examined using Next Generation Impactor (NGI). Figure 4 illustrates the deposition patterns of freeze-dried and spray-dried powders across the NGI stages. The freeze-dried formulations exhibited a predominant accumulation in the induction port, with minimal distribution across NGI stages 1 to 7. In contrast, the spray-dried formulations demonstrated a significantly greater deposition in the NGI stages beyond the pre-separator, with particularly pronounced deposition in stages 2, 3, and 4 (*p* < 0.05) when compared to the freeze-dried powders.

According to British Pharmacopoeia, NGI stages 2, 3, and 4 correspond to aerodynamic cut-off diameters of 4.46 µm, 2.82 µm, and 1.66 µm, at a flow rate of 60 L/min. This deposition pattern indicates that spray-dried powders possess a lower aerodynamic diameter relative to freeze-dried powders, enhancing their potential for deep lung deposition.

Extensive literature has highlighted the potential of spray-drying as a technique for producing inhalable powders with enhanced pulmonary delivery efficiency [10,26,28]. The superior deposition of spray-dried powders in the lower NGI stages may be attributed to their distinct physicochemical characteristics, particularly their smooth spherical morphology and low densities, which improves powder flowability in comparison to freeze-dried formulations.

**Figure 5:**
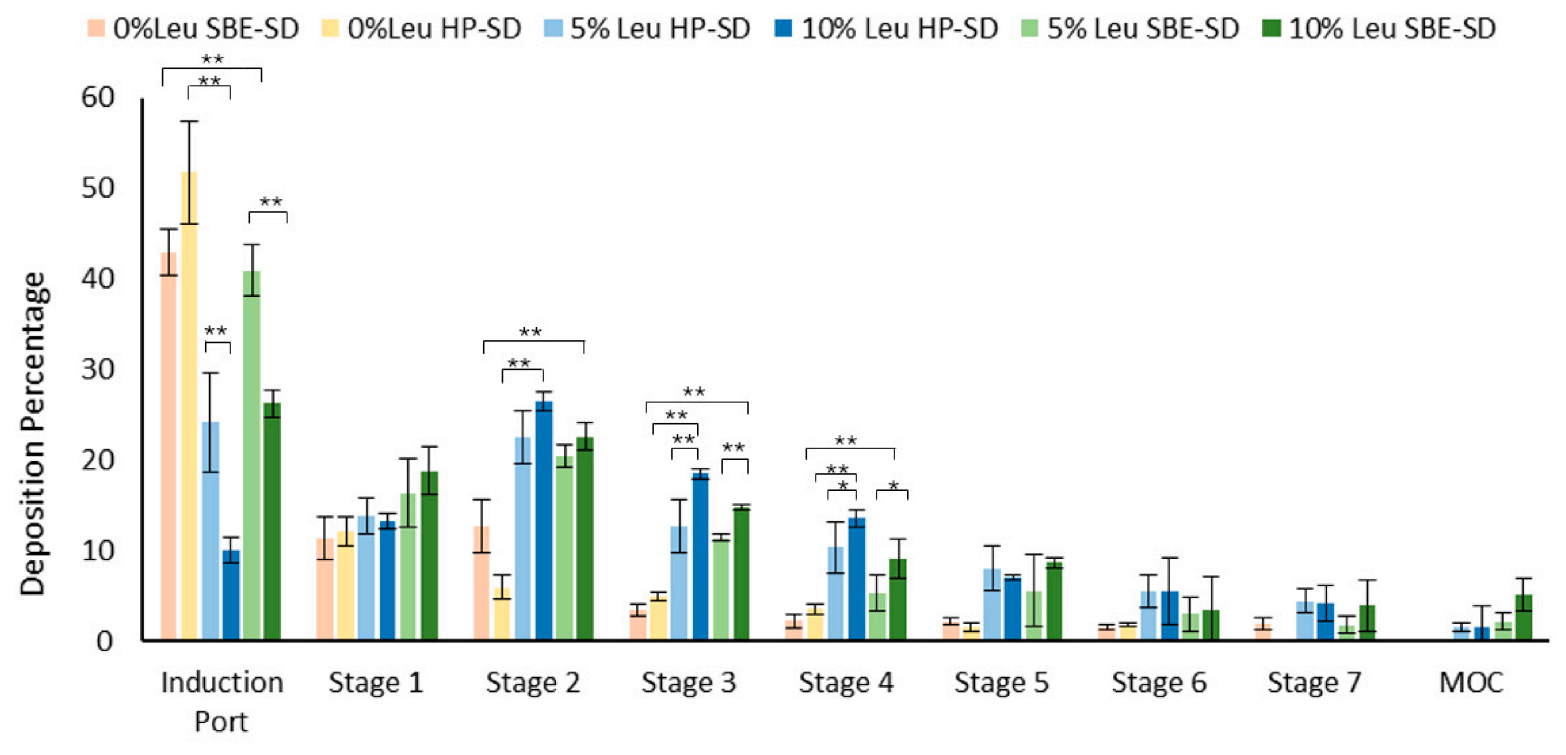
Deposition percentages of leucine-free 5% Leu SBE-SD, 10% Leu SBE-SD, 5% Leu HP-SD, and 10% Leu HP-SD formulations across NGI stages at 60 L/min (mean ± SD, n = 3). * *p* < 0.05; ** *p* < 0.01.

Figure 5 presents the deposition profiles of spray-dried cyclodextrin formulations containing 0%, 5%, and 10% leucine. The inclusion of leucine resulted in a significant reduction in powder deposition within the induction port. Specifically, for HP formulations, deposition decreased from 36.56 ± 1.75% to 24.16 ± 1.44% for 5% leucine and further to 9.99 ± 1.44% with 10% leucine. A similar trend was observed for SBE formulations, where deposition was reduced from 51.67 ± 1.40% to 40.92 ± 2.91% and 26.18 ± 1.43% with the incorporation of 5% and 10% leucine, respectively. The aerodynamic cut-off diameter of the induction port ranges from 10 to 14 µm, depending on the flow rate, indicating that particles depositing in this region are not considered respirable and are likely to settle in the oropharyngeal and laryngeal regions [29].

The deposition of spray-dried powders across NGI stages was significantly enhanced with the incorporation of both 5% and 10% leucine for HP-SD and SBE-SD formulations. Figure 4 illustrates that leucine-free spray-dried powders were deposited only up to stage 6 for HP-SD and stage 5 for SBE-SD. In contrast, spray-dried powders containing leucine demonstrated extended deposition across all NGI stages up to the MOC stage. This enhancement in dispersion efficiency may be attributed to modifications in particle morphology and density resulting from leucine incorporation.

As discussed above, leucine-containing spray-dried powders exhibited corrugated surfaces with smaller particles embedded within larger aggregates, leading to improved dispersibility throughout the NGI. The literature further supports that leucine addition enhances the in vitro aerosolisation properties of spray-dried formulations, thereby increasing their deposition across NGI stages [29,30].

Furthermore, during spray drying, leucine may preferentially accumulate at the droplet surface and form a leucine-enriched outer layer. This surface modification can reduce particle surface energy and the effective contact area between neighbouring particles. Consequently, the overall cohesive interaction between particles, including the effective contribution of van der Waals forces, may be reduced, facilitating deagglomeration during aerosolisation. The corrugated and hollow particle morphology observed following leucine incorporation may further reduce particle–particle contact points and limit powder aggregation. In addition, the relatively hydrophobic leucine-enriched surface may reduce moisture adsorption and minimise moisture-induced particle bridging or agglomeration, thereby helping to preserve dispersibility during storage [31,32].

The impact of leucine concentration on the aerodynamic particle size was statistically significant. The deposition in NGI stages 2, 3, and 4 was significantly higher for formulations containing 10% leucine compared to those with 5%, corresponding to cut-off aerodynamic diameters of 4.46 µm, 2.82 µm, and 1.66 µm, respectively. Consequently, powders formulated with 10% leucine exhibited a smaller aerodynamic diameter than those containing 5%, indicating an increased likelihood of deposition within the small conducting airways.

A comparative analysis of deposition between the two cyclodextrin-based formulations following leucine incorporation revealed that HP-Zn(DDC)_2_ complexes exhibited significantly greater deposition across NGI stages at both 5% and 10% leucine concentrations compared to SBE-Zn(DDC)_2_ formulations. This suggests that HP-Zn(DDC)_2_ complexes have a lower aerodynamic diameter, which may be attributed to their lower bulk density, as previously reported.

The aerosol properties of both freeze-dried and spray-dried formulations were evaluated based on NGI deposition data obtained at a flow rate of 60 L/min (Table 3). Fine particle fraction (FPF) is a critical parameter influencing the bioavailability of inhaled drugs in vivo, as it is directly proportional to the fraction of the drug reaching the lungs. A higher FPF percentage correlates with deeper pulmonary deposition [33]. As shown in Table 3, the FPF increased significantly from 20.03 ± 2.79% to 40.12 ± 0.60% for HP-FD and HP-SD, respectively, and from 20.91 ± 4.45% to 31.76 ± 2.10% for SBE-FD and SBE-SD, respectively. These findings indicate that the spray-drying technique significantly enhances the FPF of the formulations compared to freeze-dried powders. Similar observations were reported for tobramycin, where the FPF increased from 36 ± 6% to 49.3 ± 0.8% following spray-drying.

For spray-dried powders incorporating leucine, the emitted dose (ED) and fine particle dose (FPD) were included to provide a mass-based assessment of aerosol performance. ED ranged from 7.01 ± 0.24 to 9.36 ± 0.97 mg, while FPD ranged from 2.04 ± 0.27 to 7.06 ± 0.93 mg. Overall, the leucine-containing formulations showed higher ED and FPD values than the corresponding leucine-free powders, indicating that leucine improved not only the percentage fine particle fraction but also the absolute respirable mass available for lower-airway delivery. The FPF percentage increased significantly for Zn(DDC)_2_-CD complexes. The FPF for HP-SD formulations increased from 40.12 ± 0.60% to 64.51 ± 2.96% and further to 76.77 ± 1.18% with the incorporation of 5% and 10% leucine, respectively (Table 3). A similar trend was observed for SBE-SD formulations, where the FPF increased from 31.76 ± 2.10% to 51.36 ± 2.05% and 63.60 ± 2.59% upon the addition of 5% and 10% leucine, respectively. The inclusion of leucine significantly enhanced the aerosolisation properties of the formulations.

The increase in FPF can be attributed to the lower powder densities and the surface corrugation induced by leucine incorporation. It has been well-reported that leucine addition improves the physicochemical properties of spray-dried powders, enhancing their suitability for pulmonary delivery. The inclusion of leucine in spray-dried fisetin–SBE cyclodextrin formulations for NSCLC treatment was reported to significantly improve the aerosolisation properties of the formulation. Similarly, leucine incorporation into spray-dried poly-L-lysine formulations for pulmonary infection treatment resulted in powders with improved inhalation characteristics [34].

Powder density plays a crucial role in lung deposition, with an established inverse correlation between bulk density (ρb) and FPF [35]. The observed increase in FPF for spray-dried formulations is likely associated with their lower bulk density. A decrease in powder density corresponded with increased deposition in NGI stages. These findings align with previous reports by crucial and Ahsan (2011) [36] and Chvatal et al. (2019) [37], which demonstrated that lower-density powders exhibit superior in vitro aerosolisation properties. Additionally, Zhao et al. (2018) [38] found that cyclodextrin-based dry powder inhaler (DPI) formulations resulted in powders with reduced densities and consequently improved FPF values.

The Mass Median Aerodynamic Diameter (MMAD) is a key parameter for characterising the aerodynamic behaviour of respirable particles, representing the diameter at which 50% of the aerosolised drug mass lies below the given threshold. Spray-dried formulations exhibited significantly lower MMAD values compared to freeze-dried powders, indicating improved suitability for pulmonary deposition. However, the addition of leucine at 5% and 10% concentrations did not significantly alter the MMAD or geometric standard deviation (GSD) values of the spray-dried powders for either cyclodextrin formulation. These findings are consistent with previous studies, which reported that leucine incorporation into spray-dried powders did not influence MMAD and GSD values [39].

Overall, Zn(DDC)_2_-CD complexes formulated with leucine exhibit significant potential for pulmonary drug delivery. The enhanced aerosolisation properties, including increased FPF and reduced powder density, indicate a high likelihood of deposition in the lungs while minimising oropharyngeal deposition. This targeted delivery mechanism may contribute to localised therapeutic effects while reducing systemic absorption and associated side effects.

## 4. Conclusions

This study successfully developed and characterised inhalable dry powder formulations of Zn(DDC)_2_–cyclodextrin complexes for potential pulmonary delivery. Spray-dried formulations showed superior aerodynamic performance, flowability, and lung deposition potential compared with freeze-dried formulations. Leucine incorporation further improved aerosolisation, with 10% leucine formulations achieving the highest fine particle fraction and respirable fraction, supporting their potential for deep lung deposition.

SEM analysis confirmed that spray-dried powders had a more uniform spherical morphology, while leucine-modified formulations showed increased surface roughness, likely improving dispersibility. Density, flowability, NGI, and TSI data further supported the suitability of spray-dried formulations for DPI application. Among the tested formulations, 10% leucine HP-SD showed the most favourable overall performance, with a bulk density of 0.13 ± 0.09 g/cm^3^, FPF of 76.77 ± 1.18%, RF of 76.77 ± 1.18%, MMAD of 2.83 ± 0.46 µm, emitted dose of 9.36 ± 0.97 mg/run, and fine particle dose of 7.06 ± 0.93 mg/run. These findings support its selection as the lead candidate for further preclinical evaluation.

Overall, Zn(DDC)_2_–cyclodextrin dry powder inhaler formulations show promise as a localised therapeutic approach for NSCLC. However, this study was limited to physicochemical and in vitro aerodynamic characterisation, and no cell-based efficacy, normal lung-cell tolerability, in vivo pharmacokinetic, anti-tumour efficacy, or long-term stability studies were performed.

Future work should assess Zn(DDC)_2_ dissolution and release from the lead HP-β-CD and SBE-β-CD formulations in biorelevant simulated lung fluid, including the effect of leucine concentration on dissolution kinetics and drug availability after aerosol deposition. Further studies should evaluate pulmonary tolerability using normal lung epithelial cell models, inflammatory-response assays, and, where appropriate, in vivo safety assessment. Formal moisture-sorption and stability studies under controlled temperature and relative-humidity conditions, including ICH-relevant storage conditions, are also required to assess hygroscopicity, solid-state stability, Zn(DDC)_2_ content, aerosolisation performance, and recrystallisation risk. In vivo studies should further determine lung tissue distribution, retention, systemic exposure, pharmacokinetic behaviour, pulmonary tolerability, and anti-tumour efficacy in an appropriate NSCLC model.

## Figures and Tables

**Table 1:** Bulk and tapped densities of freeze-dried, spray-dried, and leucine-containing Zn(DDC)_2_-cyclodextrin complexes (mean ± SD, n = 3). * *p* < 0.05 compared with freeze-dried formulations.

Formulation	Tapping Density (g/cm^3^)	Bulk Density (g/cm^3^)
HP-FD	2.04 ± 0.91	0.37 ± 0.06
SBE-FD	2.55 ± 0.73	0.41 ± 0.09
HP-SD	* 1.03 ± 0.71	* 0.23 ± 0.07
SBE-SD	* 1.35 ± 0.63	* 0.31 ± 0.02
5% Leu HP-SD	* 0.94 ± 0.12	* 0.18 ± 0.01
10% Leu HP-SD	* 0.72 ± 0.34	* 0.13 ± 0.09
5% Leu SBE-SD	* 1.05 ± 0.02	* 0.34 ± 0.04
10% Leu SBE-SD	* 0.91 ± 0.34	* 0.28 ± 0.01

**Table 2:** Powder flowability of freeze-dried, spray-dried, and leucine-containing Zn(DDC)_2_-cyclodextrin complexes, measured using a laser beam method (mean ± SD, n = 3). * *p* < 0.05.

Formulation	Powder Flowability (g/s)		
	Nozzle Size		
	25 mm	15 mm	10 mm
HP-FD	15.2 ± 2.1	-	-
SBE-FD	19.5 ± 2.3	-	-
HP-SD	20.4 ± 1.4	15.9 ± 0.9	9.7 ±1.4
SBE-SD	19.0 ± 2.1	13.8 ± 2.4	10.3 ± 1.6
5% Leu HP-SD	* 25.7 ± 2.3	* 18.3 ± 1.3	* 10.4 ± 2.1
10% Leu HP-SD	* 28.1 ± 1.8	* 21.7 ± 1.8	* 11.0 ± 0.9
5% Leu SBE-SD	* 23.9 ± 1.6	* 16.0 ± 1.2	* 12.4 ± 1.9
10% Leu SBE-SD	* 27.4 ± 2.4	* 20.1 ± 0.8	* 15.3 ± 1.3

**Table 3:** Aerosolisation properties of freeze-dried, spray-dried, and leucine-containing powders calculated from NGI data at 60 L/min (mean ± SD, n = 3). * *p* < 0.05; ** *p* < 0.01; *** *p* < 0.001.

Formulation	FPF %	ED (mg)	FPD (mg)	MMAD (μm)	GSD (μm)	RF %
HP-FD	20.03 ± 2.79	7.01 ± 0.24	2.88 ± 0.35	* 4.63 ± 0.23	* 2.33 ± 0.11	18.50 ± 2.79
SBE-FD	20.91 ± 4.45	7.59 ± 1.42	2.04 ± 0.27	* 3.16 ± 0.05	1.78 ± 0.07	20.91 ± 1.45
HP-SD	** 40.12 ± 0.60	7.96 ± 0.81	* 4.01 ± 0.08	3.13 ± 1.12	1.73 ± 0.05	* 37.38 ± 5.06
SBE-SD	** 31.76 ± 2.10	8.10 ± 0.12	3.78 ± 1.29	2.33 ± 0.05	1.63 ± 0.12	* 31.22 ± 4.28
5% Leu HP-SD	*** 64.51 ± 2.96	* 9.04 ± 1.36	** 5.66 ± 0.81	2.96 ± 0.32	1.56 ± 0.05	** 64.51 ± 2.96
10% Leu HP-SD	*** 76.77 ± 1.18	* 9.36 ± 0.97	*** 7.06 ± 0.93	2.83 ± 0.46	1.63 ± 0.07	** 76.77 ± 1.18
5% Leu SBE-SD	** 51.36 ± 2.05	* 9.06 ± 0.17	4.89 ± 0.30	2.30 ± 0.01	1.62 ± 0.04	** 42.60 ± 2.32
10% Leu SBE-SD	*** 63.60 ± 2.59	* 9.14 ± 1.82	* 5.17 ± 0.74	2.16 ± 0.05	1.67 ± 0.05	** 56.72 ± 1.46

## Data Availability

The data presented in this study are available on request from the corresponding authors.
